# A Meta-Analysis on the Effectiveness of Sertaconazole 2% Cream Compared with Other Topical Therapies for Seborrheic Dermatitis

**DOI:** 10.3390/jpm12091540

**Published:** 2022-09-19

**Authors:** Simona Roxana Georgescu, Cristina Iulia Mitran, Madalina Irina Mitran, Andreea Amuzescu, Clara Matei, Mircea Tampa

**Affiliations:** 1 Department of Dermatology, “Carol Davila” University of Medicine and Pharmacy, 020021 Bucharest, Romania; 2 Department of Dermatology, “Victor Babes” Clinical Hospital for Infectious Diseases, 030303 Bucharest, Romania; 3 Department of Microbiology, “Carol Davila” University of Medicine and Pharmacy, 020021 Bucharest, Romania

**Keywords:** seborrheic dermatitis, sertaconazole, *Malassezia*, therapy

## Abstract

Seborrheic dermatitis (SD) is a relapsing inflammatory skin disorder that affects the seborrheic areas of the body. Its etiology is not completely elucidated; however, the link between disease exacerbations and the proliferation of *Malassezia* spp., along with the good response to antifungal agents, indicate the role of fungi in its pathophysiology. Sertaconazole nitrate is a relatively new imidazole antifungal agent with a particular structure, consisting in a benzothiophene ring similar to the indole ring of tryptophan, and it acts mainly through the inhibition of ergosterol synthesis and the formation of pores in the fungal cell membrane. The aim of our study was to evaluate the efficiency of sertaconazole 2% cream compared with other topical treatments in patients with SD. We performed an extensive literature search by browsing the PubMed database with the keyword combination “sertaconazole AND seborrheic dermatitis AND clinical trial”, which retrieved eight controlled clinical trials evaluating the effects of sertaconazole in SD. All of the clinical trials included a standard scoring index (SI). At 28 days since the beginning of the treatment, the sertaconazole regimen was associated with a significantly higher percentage of patients with mild SI and a lower percentage of patients with moderate or severe SI (odds ratio 0.51) than the other investigated treatments—hydrocortisone, ketoconazole, clotrimazole, metronidazole, pimecrolimus, and tacrolimus (odds ratio 1.95). In conclusion, treatment with sertaconazole 2% cream may represent an efficient alternative therapy for patients with SD.

## 1. Introduction

Seborrheic dermatitis (SD) is a chronic, recurrent inflammatory condition that affects approximately 1–3% of individuals worldwide. Its pathogenesis involves an interplay between several factors, such as genetic predisposition, altered skin barrier, excessive secretion of sebaceous glands, skin microbiome, and immune response [1,2]. A systematic review that analyzed the genetic basis of SD identified 11 gene mutations or protein deficiencies that induced an SD or SD-like phenotype in humans or mice. The majority of encoded proteins are involved in epidermal differentiation or immunity [1]. The innate immune response seems to play a critical role in the pathogenesis of SD. Individuals with impaired immunity, such as HIV-infected patients, transplant recipients, and patients with alcoholic pancreatitis or various neoplasms, are more susceptible to develop SD. Several neurologic and psychiatric disorders, including Parkinson's disease, Alzheimer’s disease, syringomyelia, epilepsy, cerebrovascular infarcts, etc., are also related to SD [3]. SD is more common in men, and its onset is more frequent at puberty; therefore, hormones, especially androgens, could play a role in its pathogenesis [4]. In recent years, the role of oxidative stress has been highlighted in many skin diseases, including SD [5,6,7,8,9]. 

A special role in the pathogenesis of seborrheic dermatitis is attributed to *Malassezia* spp. The genus *Malassezia* belongs to the phylum *Basidiomycota* and includes 17 species. *Malassezia* spp. normally colonize the skin of healthy individuals, but under appropriate conditions, the yeasts have the ability to invade the stratum corneum and can interact directly or indirectly with keratinocytes, melanocytes, and immune cells in the epidermis [10]. Louis-Charles Malassez was the first researcher to hypothesize that fungi are involved in the pathogenesis of SD in 1874 [11]. Eight species of *Malassezia* have been isolated from human skin [12]. *M. globosa* and *M. restricta* were most commonly isolated from patients with SD [13]. *Malassezia* spp. develop favorably in hair follicle and sebum-rich areas of the body [14].

Alterations of the skin barrier function in individuals with a genetic predisposition, along with sebum composition abnormalities, create a suitable environment for the overgrowth of *Malassezia* spp. [15]. *Malassezia* spp. are lipophilic yeasts able to secrete lipases that lead to the formation of fatty acids, such as oleic acid and arachidonic acid, and lipid peroxides that generate an inflammatory response, epidermal proliferation, and disruption of the skin barrier [16]. *Malassezia* spp. promote dendritic cell maturation and stimulate Th2 cells, inducing the activation of numerous inflammatory pathways and the release of a plethora of proinflammatory cytokines. Alteration of keratins 1, 10, and 11 and ceramides, which are important epidermal structural components, was also highlighted in SD [16]. Data from an in vitro study showed that *Malassezia furfur* isolated from SD lesions produced significantly more bioactive indolic substances compared with strains isolated from the healthy skin [10]. Indolo [3,2-b] carbazoles can cross the epidermis, reach the spinous and granular layers, and stimulate the aryl hydrocarbon receptor, a process that has been associated with the differentiation of Th17 cells and inflammation in transgenic mice [4].

However, the level of *Malassezia* does not correlate with the severity of the disease. The alteration of bacterial diversity seems to be a better predictor [17]. The skin microbiome can contribute to the proliferation of *Malassezia* through the hydrolysis of sebum, and in this way the required nutrients for fungal growth are provided. Thus, a predominance of *Acinetobacter*, *Staphylococcus,* and *Streptococcus* spp. on lesional skin was observed [18]. In patients with SD, a decreased level of *Cutibacterium* spp. was identified, demonstrating the role of this microorganism in maintaining a balance between microbial communities [2]. Recent studies have shown that fungi such as *Candida* and *Aspergillus* are also overexpressed in patients with SD [19].

The role of *Malassezia* yeasts in the occurrence of SD is supported by the effectiveness of antifungal treatments and by the decrease in *Malassezia* cell amount in patients who experience symptom relief [20]. Sertaconazole is an imidazole antifungal agent that inhibits the biosynthesis of ergosterol, an essential element of the fungal cell wall, and in addition binds to nonsterol lipids in the cell membrane, leading to impaired cell function [21]. We conducted a meta-analysis to investigate the efficacy of sertaconazole 2% cream in the treatment of SD compared with other topical therapies.

## 2. Materials and Methods

**Figure 1:**
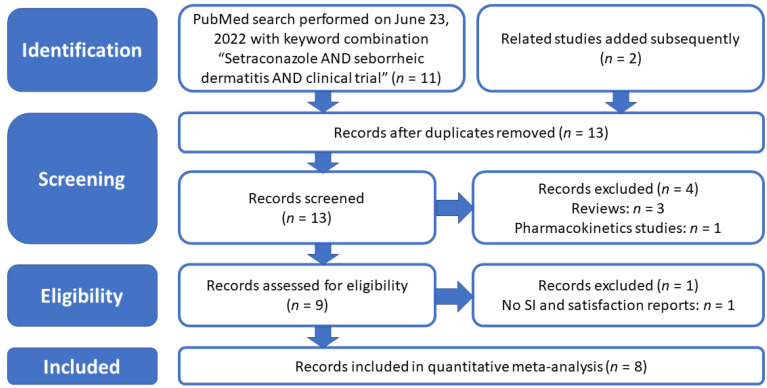
Retrieval and selection of publications to be included in the meta-analysis according to the PRISMA guidelines.

We performed a web-based search of the PubMed medical literature database (https://pubmed.ncbi.nlm.nih.gov accessed on 23 June 2022) with a specific combination of search terms: “Sertaconazole AND seborrheic dermatitis AND clinical trial”. This search retrieved 11 results, supplemented with another two papers retrieved by searching the “Similar articles” section displayed in PubMed when accessing a search hit. From this initial group of publications, we excluded two review papers, one paper addressing the pharmacokinetics and side effects of different sertaconazole nitrate formulations, and in the eligibility assessment phase, one paper that did not report adequate quantitative data, such as the scoring index (SI) assessment of treatment effects, as recommended by Koca et al. [22]. The selection procedure of the studies included in this meta-analysis was compatible with the principles and guidelines of PRISMA (Preferred Reporting Items for Systematic Re-views and Meta-Analyses) [23,24], and the consecutive selection steps are illustrated in Figure 1. 

The selected and excluded studies were uploaded to a meta-analysis project created in Review Manager (RevMan) Version 5.4.1, The Cochrane Collaboration, 2020 [25]. Subsequently, we performed three quantitative comparisons for dichotomous variables (frequencies of certain outcomes in experimental vs. control group), with the same software package, selecting a Cochran–Mantel–Haenszel model with fixed effect and computation of odds ratios (OR) as a measure of effect, and a 95% confidence interval. 

## 3. Results

For the eight studies finally included in the meta-analysis, listed in Table 1, as quantitative variables for comparison, we retrieved the scoring indexes (SI) at different time intervals over the duration of treatment (28 days), particularly the frequencies of mild SI and cumulated frequencies of moderate or severe SI in the treatment (sertaconazole) vs. control (other treatments) groups, as well as the frequencies of “good” rankings in the patient satisfaction questionnaires administered at the end of treatment (28 days). 

**Figure 2:**
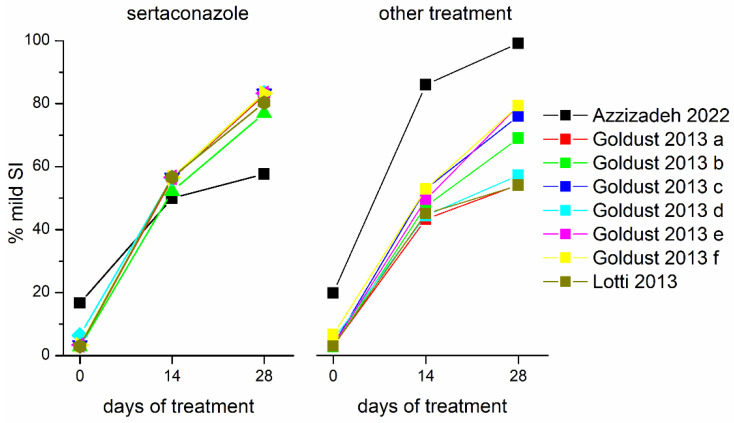
Dynamics of percentages of patients featuring mild SI at different times (0, 14, and 28 days of treatment) in the sertaconazole vs. control groups in the eight studies included in the meta-analysis.

The dynamics of the percentages of patients included in the mild SI groups at different moments of time (beginning of treatment, 14 days of treatment, and 28 days of treatment), illustrated in Figure 2, suggest differences in treatment outcomes between the sertaconazole group and control group, receiving a different treatment. 

**Figure 3:**
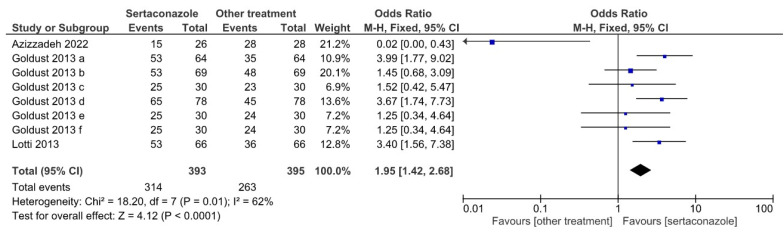
Forrest plot illustrating the effect of sertaconazole vs. other treatments in the eight selected studies for the mild SI at 28 days of treatment (OR with 95% confidence interval, heterogeneity, and overall effect).

Figure 3 represents a Forrest plot showing the OR of sertaconazole vs. other treatments in the eight selected studies for the mild SI at 28 days of treatment. The cumulated number of patients was 393 in the sertaconazole groups and 395 in the other treatment groups; the OR was 1.95, favoring sertaconazole (95% confidence interval of 1.42–2.68), the heterogeneity test yielded a χ^2^ value of 18.20 (*p* = 0.01), and the overall effect yielded a *z* of 4.12 (*p* < 0.0001). 

**Figure 4:**
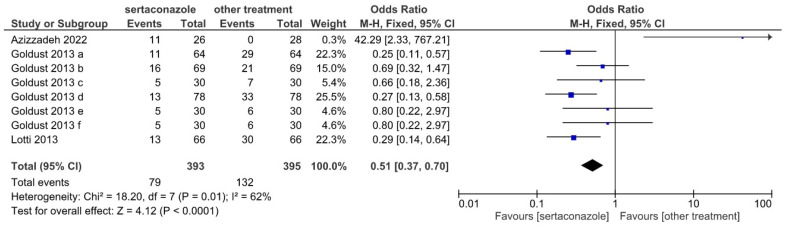
Forrest plot illustrating the effect of sertaconazole vs. other treatments in the eight selected studies for the moderate and severe SI at 28 days of treatment (OR with 95% confidence interval, heterogeneity, and overall effect).

Complementary, the cumulated frequency of moderate or severe SI in the sertaconazole vs. other treatments groups (Forrest plot shown in Figure 4) yielded an OR of 0.51 (95% confidence interval 0.37–0.70), again favoring sertaconazole. The χ^2^ value was 18.20 (*p* = 0.01) and the overall effect featured a *z* of 4.12 (*p* < 0.0001). 

**Figure 5:**
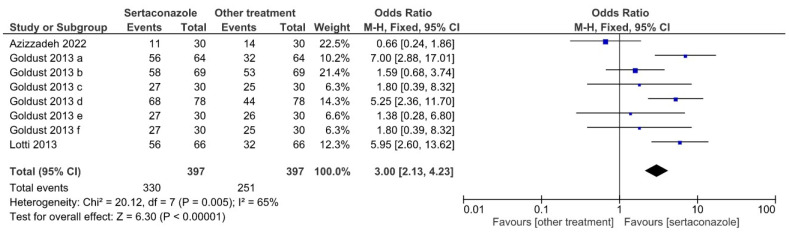
Forrest plot illustrating the effect of sertaconazole vs. other treatments in the eight selected studies for the “good” ranking in the self-assessment and patient satisfaction questionnaire at 28 days of treatment (OR with 95% confidence interval, heterogeneity, and overall effect).

Figure 5 illustrates a Forrest plot for the analysis of frequencies of patients indicating a “good” ranking in the satisfaction self-assessment questionnaire administered at 28 days of treatment; the OR was 3.00 (95% confidence interval 2.13–4.23), again favoring sertaconazole vs. other treatments. The heterogeneity test yielded a χ^2^ value of 20.12 (*p* = 0.005), and the overall effect a *z* of 6.30 (*p* < 0.00001).

## 4. Discussion

Seborrheic dermatitis is a chronic inflammatory disease with a pathogenesis closely related to the abnormal proliferation of fungi on the skin [34]. Thus, antifungals are considered first-line treatments. The most widely used topical antifungals are azoles, with very good results being obtained, especially in the case of topical ketoconazole 2%. Ciclopirox and terbinafine are other antifungals that can be used [35]. Topical corticosteroids are prescribed in SD for their anti-inflammatory effect, but patients must be carefully monitored considering the well-known adverse effects (thinning of the skin or telangiectasia), which occur especially with long-term uninterrupted use [15]. Therefore, low- to mid-potency corticosteroids are recommended. Calcineurin inhibitors (pimecrolimus and tacrolimus) can be employed to avoid the adverse effects of corticosteroids [36]. A single-blinded, randomized controlled trial that evaluated the effectiveness of hydrocortisone 1% ointment compared with tacrolimus 0.1% ointment found similar results [37]. An analysis of topical medication with an anti-inflammatory effect for SD, including studies published until September 2013, showed that there are no significant differences between corticosteroids and calcineurin inhibitors in the short term. Additionally, the results were similar in terms of their total clearance, when the group treated with corticosteroids was compared with the group treated with azoles [38]. A recent clinical trial revealed that tacrolimus 0.1% ointment is more effective than ciclopiroxolamine 1% cream for maintenance therapy in patients with a severe form of SD involving the face [39]. Okokon et al. analyzed the effectiveness of topical antifungals in SD and obtained sufficient data only in the case of ketoconazole, ciclopirox, and bifonazole. They concluded that ketoconazole and ciclopirox showed similar results regarding the alleviation of symptoms in SD. The effectiveness of ketoconazole was similar to that of topical corticosteroids, but with fewer adverse effects. The tolerability of bifonazole was lower compared with the placebo [40]. The systemic treatment is reserved for severe cases and includes itraconazole, terbinafine, fluconazole, ketoconazole, pramiconazole, prednisone, and isotretinoin. Most of the available studies focused on evaluating the effectiveness of oral antifungals [41].

Sertaconazole nitrate is a relatively new imidazole antifungal agent with a particular structure, consisting of a benzothiophene ring similar to the indole ring of tryptophan [42]. It exhibits a broad-spectrum antifungal activity against dermatophytes and yeasts, opportunistic filamentous fungi, and Gram positive bacteria [29]. The clinical efficacy of sertaconazole has been demonstrated in various skin mycoses, including candidiasis, dermatophytosis, and pityriasis versicolor [43]. Topical administration is safe, systemic absorption is low, and the cutaneous retention time is high. Sertaconazole has the advantage of being available in a variety of pharmaceutical forms (cream, gel, solution, powder, and shampoo) [44]. Depending on its concentration and the microorganism involved, sertaconazole has both a fungistatic and a fungicidal effect. The antifungal agent selectively inhibits the biosynthesis of ergosterol acting on fungal 14α-demethylase, which favors the accumulation of 14-a-methylated sterols, and subsequently cell growth and replication are dysregulated. It also has the ability to insert into the fungal cell membrane by binding to non-sterol lipids, resulting in an alteration of membrane permeability and leakage of the intracellular components [45].

Liebel et al. showed that sertaconazole inhibits the release of pro-inflammatory cytokines from activated lymphocytes in animal models of irritant contact dermatitis. Compared with other antifungal agents, the best anti-inflammatory effect was observed for sertaconazole. Only fluconazole and sertaconazole inhibited neurogenic inflammation, suggesting the possible role of these antifungal agents in the relief of itching [46]. In addition, sertaconazole exhibits anti-inflammatory and anti-itch effects through the activation of the p38-COX-2-PGE2 pathway in keratinocytes and human peripheral blood mononuclear cells [47,48]. Saki et al. revealed that sertaconazole was superior to hydrocortisone for improving the total score of disease in patients with atopic dermatitis. However, there were no differences in terms of pruritus improvement [49]. 

Elewski et al. conducted an open-label study that evaluated sertaconazole nitrate 2% cream in SD patients, and showed that it was effective with a good safety profile and improved scaling, erythema, induration, and pruritus [50]. Our meta-analysis included eight studies evaluating the efficacy of sertaconazole 2% cream in SD in comparison with the following treatments: hydrocortisone 1% cream (2 studies), ketoconazole 2% cream (one study), clotrimazole 1% cream (one study), calcineurin inhibitors (pimecrolimus 1% cream, two studies, and tacrolimus 0.03% cream, one study), and metronidazole gel 1% (one study). All studies highlighted a higher level of satisfaction 28 days after the initiation of treatment in the case of patients treated with sertaconazole, with the exception of the study conducted by Azzizadeh [26]. One month after stopping the treatment, no relapses were reported, regardless of the therapy administered. With respect to the level of satisfaction at 28 days, greater differences were observed when sertaconazole was compared with antifungals (clotrimazole and ketaconazole) than when it was compared with hydrocortisone or calcineurin inhibitors. Azizzadeh et al. noticed a significantly better improvement in SD in the case of patients treated with pimecrolimus 1% compared with those treated with sertaconazole 2%. In addition, relapses and adverse effects were less frequent in the group of patients treated with pimecrolimus 1%. The authors concluded that pimecrolimus 1% led to a faster improvement in symptoms than sertaconazole [26].

All studies included in the present meta-analysis evaluated the severity of the disease by the Scoring Index (SI), as recommended by Koca et al. The score is based on the evaluation of erythema, desquamation, itching, and irritation of each area, which is graded from zero to three (nonexistence = 0, mild = 1, moderate = 2, severe = 3). The sum of the scores was considered as the SD rank (classified into three ranges: mild (0–4), moderate (5–8), and severe (9–12)). At 14 days since the beginning of the treatment, no significant differences were observed between patients treated with sertaconazole and those treated with other topical treatments. At 28 days since the beginning of the treatment, the sertaconazole regimen was associated with a significantly higher percentage of patients with mild SI and a lower percentage of patients with moderate or severe SI (odds ratio 0.51) than the other investigated topical treatments (odds ratio 1.95).

This research has some limitations. The number of studies analyzed was small and the studies included reduced numbers of patients. In addition, none of the studies followed up the patients for more than one month; therefore, the data on relapses are inconclusive. Medium-/long-term studies are needed to establish if the disease-free interval is greater with one drug than with another.

## 5. Conclusions

The presented studies support the effectiveness of sertaconazole in SD therapy. Sertaconazole treatment was associated with a high level of satisfaction 28 days after the initiation of therapy. Additional studies including larger numbers of patients are needed to validate these results.

## Figures and Tables

**Table 1:** Main features of the clinical trials included in the present meta-analysis.

Study	Double Blinded	Rando-Mized	Groups	Mean Age	Sex	Kind of Lesion	Treatment
Azizzadeh et al., 2022 [26]	Y	Y	pimecrolimus 1% cream vs. sertaconazole 2% cream	27.5	M/F	-	twice a day for four weeks
Goldust et al., 2013 a [27]	Y	Y	sertaconazole 2% cream vs. clotrimazole 1% cream	36.62 ±13.18	M/F	localized: 57% generalized: 43%	twice a day for four weeks
Goldust et al., 2013 b [28]	Y	Y	sertaconazole 2% cream vs. hydrocortisone 1% cream	36.45 ±13.23	M/F	localized: 59.4% generalized: 40.6%	twice a day for four weeks
Goldust et al., 2013 c [29]	Y	Y	sertaconazole 2% cream vs. hydrocortisone 1% cream	32.23 ±12.09	M/F	localized: 58.3% generalized: 41.7%	twice a day for four weeks
Goldust et al., 2013 d [30]	Y	Y	sertaconazole 2% cream vs. metronidazole 1% gel	32.34 ±12.56	M/F	localized: 56.4% generalized: 43.7%	twice a day for four weeks
Goldust et al., 2013 e [31]	Y	Y	sertaconazole 2% cream vs. pimecrolimus 1% cream	30.12 ±12.56	M/F	localized: 60% generalized: 40%	twice a day for four weeks
Goldust et al., 2013 f [32]	Y	Y	sertaconazole 2% cream vs. tacrolimus 0.03% cream	32.45 ±12.78	M/F	localized: 58.3% generalized: 41.7%	twice a day for four weeks
Lotti et al., 2013 [33]	Y	Y	sertaconazole 2% cream vs. ketoconazole 2% cream	42 ±14.36	M/F	localized: 36.5% generalized: 29.5%	twice a day for four weeks

## Data Availability

Not applicable.
